# Advancing research

**DOI:** 10.7554/eLife.03980

**Published:** 2014-08-13

**Authors:** Mark Patterson, Randy Schekman, Fiona M Watt, Detlef Weigel

**Keywords:** scientific publishing, publishing, eLife

## Abstract

eLife has introduced a new type of article–the Research Advance–that allows the authors of an eLife paper to publish results that build on their original research paper.

As well as establishing a venue for the publication of promising new findings in the life and biomedical sciences, eLife is also exploring ways to improve research communication. Our consultative editorial process has received a great deal of attention, as has the open-source article viewer—eLife Lens—that was developed last year. Most recently, we have introduced a new article type that has the potential to accelerate communication by allowing researchers to publish significant ‘additions’ to original research papers.

The new article format, which we have named Research Advances, is for new results that build on previously published Research Articles or Short Reports in an important way. Authors will therefore be able to report progress in their research programs rapidly and efficiently when it is judged to be a substantial addition to the original work. These contributions might use a new technique or a different experimental design to generate results that strengthen, refine or even challenge the conclusions of the original research paper.

Contributions might use a new technique or a different experimental design to generate results that strengthen, refine or even challenge the conclusions of the original research paper.

For Research Advances there is no need for extensive introductory material (which would have been covered in the original paper) or an eLife Digest. Research Advances will also tend to be short, usually with no more than 1500 words in the main text and up to four main display items (figures, tables, videos). Authors should include a Materials and methods section but otherwise have flexibility with the structure of the article. Generally, we anticipate that the editors and reviewers who assessed the original manuscript will be asked to review the Research Advance. When published, Research Advances will be clearly linked to the original research paper, and they will also be indexed and citable in their own right.

Over the past few months, eLife authors have been contacted about this idea to assess the general level of interest. A number of submissions have already been received and the first to be accepted reports an advance in a technique used in structural biology (Scheres, 2014). In the original research paper (Bai et al., 2013), the authors reported an important step forward in a technique known as electron cryo-microscopy (cryo-EM), which can be used to study the structure of biological macromolecules at near-atomic resolution. In the Research Advance Sjors Scheres—the corresponding author on the original paper—reports how this new approach to cryo-EM can be used to study the structure of smaller macromolecules, which has the potential to open the door to a much broader range of biological insights (Kuehlbrandt, 2014; Scheres, 2014).

The work of the Scheres lab is a fine example of what we are trying to achieve with Research Advances: to build this work into a full article might well have taken a great deal more time and delayed the communication of an improvement that many researchers might benefit from. Similarly, graduate students and postdoctoral fellows who are authors on a Research Advance will have their work read and cited more quickly, which will be good for their careers.

Some of the other Research Advances that are currently under review extend the original research paper by providing new biological insights, as demonstrated by the second Research Advance to be published (Lai et al., 2014). In this work, the authors have extended their study on vesicle fusion in the synaptic transmission of nerve impulses. Specifically, they have used the single-vesicle in vitro system described in the original article (Diao et al., 2012) to better understand the role of the protein complexin in this process.

It is also interesting that in some cases the specific experiments reported in the Research Advances that have been submitted were inspired by reviewers' comments about the original manuscript. In all cases, the eLife editors are maintaining a high standard for Research Advances. In keeping with the standards of Research Articles and Short Reports, we will only publish a Research Advance that is judged to be particularly promising. However, we are also looking ahead and thinking about how the concept might be extended.

One potential next step would be to allow researchers who were not authors on the original research paper to publish new results as a Research Advance linked to the original paper. This would open up the approach to much broader participation, and would raise a number of opportunities—as well as potential challenges. For example, attempts to replicate or extend a study might uncover limitations in the original work or they might substantially reinforce the initial investigation. Regardless of the direction of the new findings, it could be valuable for the research community to be made aware of how published research stands up to scrutiny, and would support current efforts to strengthen research reproducibility (Collins and Tabak, 2014).

The introduction of Research Advances is an experiment. Success will be evaluated on the basis of the take-up by authors and the reception from readers and other parts of the research communication ecosystem. As scientific publishing continues to evolve in a digital environment, we anticipate many more such experiments, so that we can learn how to adapt and maximise the potential of digital media to support science.

## References

[bib1] Bai XC, Fernandez IS, McMullan G, Scheres SH (2013). Ribosome structures to near-atomic resolution from thirty thousand cryo-EM particles. eLife.

[bib2] Collins FS, Tabak LA (2014). NIH plans to enhance reproducibility. Nature.

[bib3] Diao J, Grob P, Cipriano DJ, Kyoung M, Zhang Y, Shah S, Nguyen A, Padolina M, Srivastava A, Vrljic M, Shah A, Nogales E, Chu S, Brunger AT (2012). Synaptic proteins promote calcium-triggered fast transition from point contact to full fusion. eLife.

[bib4] Kuehlbrandt W (2014). Cryo-EM enters a new era. eLife.

[bib5] Lai Y, Diao1 J, Cipriano DJ, Zhang Y, Pfuetzner RA, Padolina MS, Brunger AT (2014). Complexin inhibits spontaneous release and synchronizes Ca^2+^-triggered synaptic vesicle fusion by distinct mechanisms. eLife.

[bib6] Scheres SHW (2014). Beam-induced motion correction for sub-MegaDalton cryo-EM particles. eLife.

